# Bibliometric Review on the Volatile Organic Compounds in Meat

**DOI:** 10.3390/foods11223574

**Published:** 2022-11-10

**Authors:** Qianlin Ni, Nicolò Amalfitano, Franco Biasioli, Luigi Gallo, Franco Tagliapietra, Giovanni Bittante

**Affiliations:** 1 Department of Agronomy, Food, Natural Resources, Animals and Environment, University of Padova, 35020 Padua, Italy; 2 Department of Food Quality and Nutrition, Research and Innovation Centre, Fondazione Edmund Mach (FEM), Via E. Mach 1, 38010 San Michele all’Adige, Italy

**Keywords:** meat flavor, meat aroma, VOC, meat odor, meat sensory traits, beef, pork, chicken, olfactometry

## Abstract

Meat flavor is an important aspect of meat quality that also influences consumer demand, and is therefore very important for the meat industry. Volatile organic compounds (VOCs) contribute in large part to the flavor of meat, and while increasing numbers of articles are published on this topic, reviews of these articles are very scarce. Therefore, our aim was to perform a bibliometric analysis of the scientific publications on VOCs in meat over the period 2000–2020. We selected 611 scientific sources from the Scopus database related to VOCs in meat (seafood excluded). The bibliometric information retrieved included journals, authors, countries, institutions, keywords, and citations. From this analysis, we drew up a list of the most important journals, authors, countries, and institutions, and the trends in VOC research on meat. We conducted a social network analysis (SNA) to identify the collaborations among the many authors and countries, and a keyword analysis to generate a network map of the authors’ keywords. We also determined which meat species were most frequently chosen as research subjects, traced the evolution of the various methods/instruments used, and explored the research tendencies. Finally, we point out the need for further research in defining meat quality, improving meat flavor, identifying adulterants, and certifying the authenticity of meat.

## 1. Introduction

Meat is a very important source for human nutrition and health. Ensuring meat quality and safety are, therefore, issues of increasing importance in today’s meat industry. 

Volatile organic compounds (VOCs) are small molecules deriving from several chemical precursors that are vaporized into the air. They are emitted from the surface of meat, so they have a strong relationship with certain properties of it. VOCs therefore have the potential to be useful tools for assessing various meat quality and safety traits, and for this reason they have recently been attracting a great deal of attention. Overall, they contribute to meat odor and affect consumer satisfaction [1]. Several studies have shown that specific individual VOCs can be biomarkers, providing important information on meat, such as differentiating species [2,3], breeds [4], and length of aging [5]. Moreover, VOC profiles can be used to assess the quality of meat with Protected Designation of Origin (PDO), Protected Geographical Indication (PGI) and Traditional Specialty Guaranteed (TSG) [6,7] certifications [6,7], and have the potential to be a reliable tool for identifying food adulteration [8].

Nowadays, many papers on meat VOCs are the result of multidisciplinary collaborations in areas such as meat safety from the microbiological point of view [9,10,11], changes in meat freshness during storage [12,13], changes in meat flavor following processing [14], the effects on meat aroma of the animals’ diet [15,16] and environmental conditions [17,18]. The research reviewed here covers works on various species, such as beef [19], pork [4], and chicken [20], as well as turkey [21], duck [22], rabbit [23], lamb and mutton [24], goat [25], yak [26], and moose [27]. Aside from fresh and cooked muscle meat, other products studied include ham [28], meat sausages [29] and burgers from different species [30], and meat substitutes [31].

Analytical technology is another important factor in this field, because capturing and identifying VOCs are complicated processes. Researchers not only use and compare techniques, such as SPME-GC-MS [19], PTR-MS [32,33], and E-nose [4], but they also study the effects of the technical characteristics of the various instruments on their results [34,35].

However, there is still very little scientific literature on this subject, so there is an urgent need to evaluate its status, limitations, and trends in order to identify future research priorities. Bibliometric analysis is one of the most useful analytical methods for objectively evaluating research quality and exploring the state of the art and hot topics in the field, and has already been used in several areas related to food quality [7,36,37]. Bibliometric analysis has two main applications: the first is descriptive, consisting of evaluating the performance of journals, authors, institutions, and countries, particularly useful for (young) researchers approaching these issues; the second consists of determining correlations using social network analysis (SNA) to gain an overall picture of the field [38], and using keyword network analysis to identify the most important topics and the relationships between them.

In this study, our aim was to analyze through a bibliometric approach, and also using a social network analysis (SNA) and a keyword analysis, the scientific articles on the VOCs of meat published in the period 2000–2020 in order to shed light on the current state of research and the tendencies in the field, and thus provide researchers with a basis for further work on improving meat flavor and meat quality, identifying adulterated meat, and authenticating meat origins. 

## 2. Materials and Methods

### 2.1. Extracting Bibliometric Information

The various steps involved in collecting and analyzing the bibliographic information were as follows:

- conduct a search of publications in the Scopus database using the keywords “Volatile organic compounds & Meat”;
- restrict the timespan to 2000–2020;
- select only English language articles;
- screen the 1482 articles retrieved to identify and exclude those not related to the aims of this work, such as those on the VOCs of fish meat or meat broth, or atmospheric pollution related to meat;
- compile a dataset of the 611 selected articles for bibliometric analysis;
- extract bibliometric information from Scopus by CVS file download, including year, journal, author(s), institution(s), country/ies, keywords, citations, etc.;
- manually complete the database to include two columns for information on “meat species” and “instrument/analytical method”.

### 2.2. Data Statistics

A quantitative statistical approach was adopted for evaluating the performances of journals, authors, institutions and countries, including the number of articles published per year, total number of citations, the most cited articles, and the most productive authors, institutions and countries. We also considered the scientific influence of journals and authors using metrics such as H-index scores, impact factors, and quartile of journal, and other indicators that were added to each entry in the dataset.

A summary of the information extracted and retained in the dataset is given in Table 1.

### 2.3. Social Network Analysis (SNA)

Social network analysis (SNA) was carried out with the “bibliometric” package in R studio, which revealed the patterns of the relationships among the data, and allowed us to make qualitative and quantitative assessments of the contributions of the variables (authors, institutions, countries) shown on the nodes and the edges [39]. For our study, the objects we picked were authors, institutions, and countries. The network mapping results were visualized with the VOSViewer software. 

We used a keywords network analysis to explore the knowledge structures among the research fields; our original bibliometric dataset included “author keywords” and “index keywords”, but for this study we used the authors’ keywords as they give a more direct indication of the research topic despite being more subjective. Analysis of the authors’ keywords reveals the patterns of topic relationships at the micro level, which can help researchers find the hot topics and trends in the field. More simple classification statistics and trends were used for the studied species and instrument information.

## 3. Results and Discussion

The earliest research into the VOCs of meat was published at the beginning of the 20th century, when [40] reported finding VOCs in beef for the first time. Over the following decades, only a few papers were published, but in the mid-20th century VOCs in meat started to gain greater attention, and during the second half of the last century about 10 articles per year were published, including some on related topics, such as meat broth, meat extract, and fish meat. By the end of the 20th century, there had been a moderate increase in published research on VOCs, but it has only been in the present century that research on the VOC profiles of meat really took off. The last five years in particular have seen an exponential increase, which seems to be due to breakthroughs in the available instruments/technologies over this period, but also due to increasing interest from consumers and the meat industry, who are now paying greater attention to meat flavor and quality, and food safety.

In this study, we focus only on this century, from the years 2000 to 2020, and exclude research on related topics.

### 3.1. Journals and Publications

As we have seen, Table 1 summarizes the information retrieved from Scopus, and some of the statistics derived from it give an initial overall picture of the data collected. Bibliometric analysis of that database allowed us to extract further information, starting with the trends in the numbers of articles published and the citations they received over the period of time studied.

**Figure 1:**
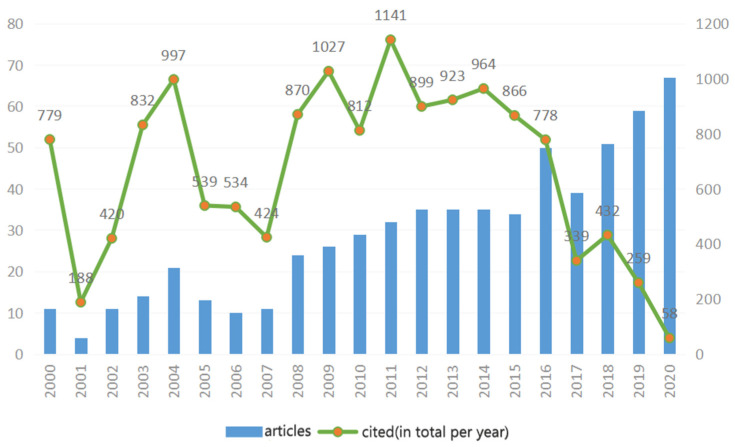
Number of articles by year of publication and number of citations received in subsequent years.

Figure 1 shows the number of articles by year of publication and the number of citations the articles received in the years following their publication (sum of total citations per year of publication). The general trend is increasing and can be divided into three periods: the first is 2000–2007, when the number of published articles varied between 5 and 20 per year; the second period is 2008–2015, which saw an increase from 25 to 35 papers per year; the third period is the last 5 years (2016–2020), when the number of publications increased from 38 to 66 per year. The total number of citations is obviously related to the number of articles published. In the first period there were about 50 citations per article, in the second period about 30 citations per article, and during the last period, as expected, the number of citations per paper dropped from about 20 per article to just a few for the most recently published, although the number of citations of these articles is, of course, expected to increase greatly over the coming years. It is also clear that the large number of citations received by the older articles is related to the large increase in articles published in subsequent years.

The conclusion that can be drawn is that there is a large and growing interest on the part of the scientific community in the VOC profile of meat, and this interest is expected to increase further in the near future. 

### 3.2. The Most Prolific Journals and Their Evaluation and Evolution

From 2000 to 2020, 98 different journals published articles on meat VOCs. Of these, 48% are ranked in the first quartile (Q1) of their highest subject category, 31% in Q2, 13% in Q3, and 8% in Q4 or unknown. The average H-index of the journals is 83, and the average impact factor 2.5. From our quantitative analysis, we obtained the ranking of the journals according to the number of articles they had published; the top 25 are listed in Table 2.

The top journal with articles (i.e., one-quarter of the total) is Meat Science, which published studies on a large variety of topics. The second is Food Chemistry, with sixty articles (one tenth of the total), whose main topics concern meat composition, metabolism pathways and comparison of methods/instruments. The next is Journal of Food Science, with 36 articles, mainly concerned with describing VOC profiles rather than with the complexities of VOC pathways.

Another aspect is the scientific influence of the journals; so, we re-ranked the journals by impact factor and H-index. The journals with the highest impact factors (10.3 to 7.1) are in the engineering fields (*Biosensors and Bioelectronics*, *Ultrasonics—Sonochemistry*, *Sensors and Actuators*, *B: Chemical*), although the number of articles published is very low. The journals with the highest H-indices are those publishing a large number of articles per year combined with a high impact factor. In first place is *Analytical Chemistry* (310), followed by *Applied and Environmental Microbiology* (310), *Journal of Agricultural and Food Chemistry* (280), and *Food Chemistry* (221).

*Meat Science* remained the leading journal throughout the 20 years. *Food Chemistry* has been in second position since 2009, while the *Journal of Food Science* gained third position in 2011, replacing the *Journal of Agricultural and Food Chemistry*. 

It is worth noting that out of the 25 top journals, 10 are published in the United States, while about one-third are published by a single publishing company (Elsevier, Amsterdam, The Netherlands).

### 3.3. Most Cited Articles

The top 25 most cited articles dealing with meat VOC profiles are listed in Table 3. 

Both of the first two papers discuss changes in the VOC profiles of beef due to spoilage by bacteria, and each had close to 200 citations at the time of retrieval. They were published in 2009 and 2015, with corresponding authors from the University of Naples Federico II, Italy, and the Agricultural University of Athens, Greece, respectively. Both research groups have strong collaborations with other groups in this field. The third article, from the Matforsk AS—Norwegian Food Research Institute, Norway, deals with the antioxidant activity of grape seed extract to prevent meat spoilage, and puts forward new ideas on non-destructive methods for monitoring meat quality (hexanal and pentanal as the VOC markers). If we look at the total citations per year, we can see that the top four articles are among the six articles with more than ten citations per year (Table 3). 

### 3.4. The Most Productive Authors and Their Collaborations 

The 611 selected articles on meat VOC profiles were authored by 1862 researchers, the 20 most productive of whom are listed in Table 4. 

In terms of number of articles, the most important research group is that of D.U. Ahn at Iowa State University (USA), who published 38 articles during this 20-year period, receiving more than 1000 citations altogether, while 17 of these articles received more than 17 citations each (H-index). Ahn and his group worked on various research topics, such as meat composition, meat spoilage caused by bacteria, and VOC pathways during processing, and mainly on pork, chicken, sausages and ham. 

The second most productive author in terms of articles published (37), but top for the number of citations received, is J.M. Lorenzo of the Centro Tecnológico de la Carne de Galicia in Spain, whose H-index was 21 (the highest) for articles on VOCs. His main research topic is the use of antioxidants in meat. 

The third most productive author is M. Flores of the Instituto de Agroquímica y Tecnología de Alimentos (IATA-CSIC) Valencia, Spain (24 articles, 651 citations and H-index 16), who works in particular on the VOC profiles of fermented meat. 

It is worth noting that half of the authors listed in Table 4 are Spanish, almost a quarter are American, and a quarter are Chinese. 

**Figure 2:**
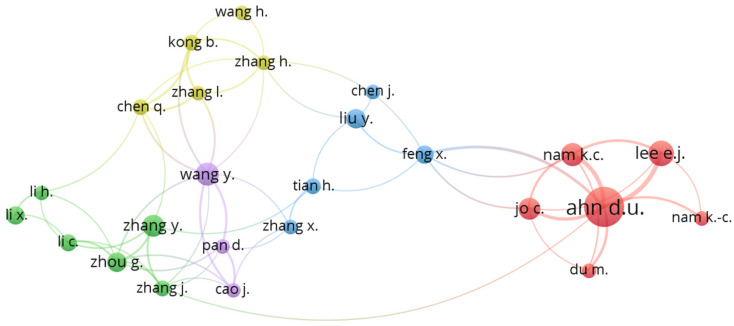
Main networks of the authors’ collaborations. The inclusion criteria were authors with more than 5 published papers and engaged in collaborations (authors working alone excluded). All together there were 88 connections, but only the 25 most closely connected are shown here. The size of the circles represents the number of papers, while the thickness of the lines represents the strength of the collaboration in terms and numbers of joint publications and their proportion of the total. Bigger circles and thicker lines indicate higher productivity and stronger collaboration between the two authors.

Having examined the authors by country, we now turn to our analysis of the networks of authors’ collaborations, represented in Figure 2.

**Figure 3:**
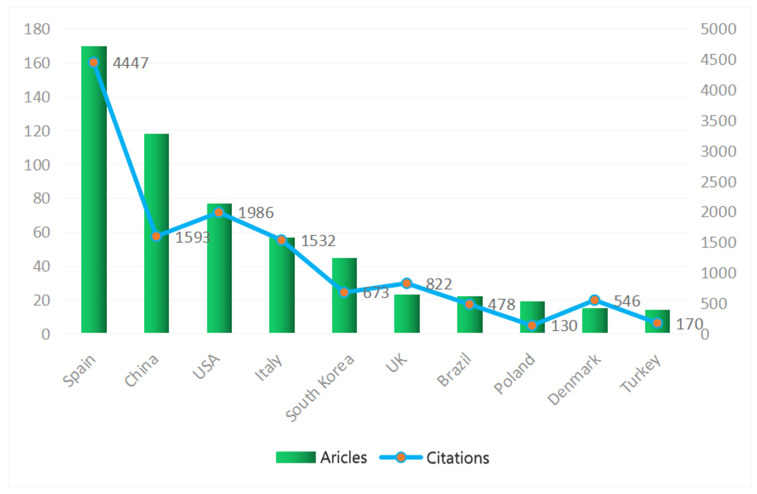
Countries of origin of the corresponding authors of articles ranked according to the number of articles they published and the number of citations they received.

The fourth author listed in Table 4, E.J. Lee, (15 articles, 264 citations and H-index 10) is one of D.U. Ahn’s close co-workers. Other authors in the leading group (Figure 3), who are involved in the most collaborations are Wang Y, Zhang Y, and Feng X, all from China, an indication of the rapid growth in this research topic in East Asia (not only China) and their broad collaborative approach. In contrast, the major European groups working on meat VOCs are clearly “stand alone” groups that do not enter into many collaborations with other groups in the same or other countries.

### 3.5. The Most Productive Countries and Institutions and Their Collaborations 

Table 5 shows the 10 most productive institutions in the research field of meat VOC profiles. 

As can be seen, the leading institution globally is Iowa State University in the USA, the only American institution in the top 10 research centers. 

Of the other nine institutions, four are in Southern Europe (Spain and Italy), and 5 are in East Asia (China, South Korea and Japan). The top Spanish institutions are two public institutes and one university, the top Italian institution is a university, and four of the five East Asian institutions are universities. Spain, China, USA, Italy and South Korea also hold leading positions among the most productive countries in terms of number of articles published and citations received (Figure 3). Between them, they account for about three quarters of all articles and citations from a total of 50 countries worldwide. The Asian countries have fewer citations per publication than the other countries, mainly because most of their articles were published in the last few years.

It is worth noting that all these leading countries are conducting basic and applied research, although a larger proportion of American research is focused on meat processing and the needs of the meat industry, while Southern European countries are more concerned with food safety, traditional products, and meat from local breeds, and Asian countries with technological issues and product characterization, including traditional foods. 

Regarding inter-country collaborations, researchers from the USA collaborate in particular with researchers from East Asia (mainly China and South Korea), whilst Southern European countries collaborate with each other, Northern European countries (especially the UK, France, the Netherlands and Poland) and Latin America (mainly Brazil and Mexico).

### 3.6. Keyword Analysis and Networks

There are two types of keywords in the bibliometric dataset, authors’ keywords and index keywords. For our study, we used authors’ keywords in order to capture the central topics of their research [65]. The original dataset contained 1447 keywords, although of course a large number of these had similar meaning, such as “Volatile compounds” and “Volatile organic compounds”, “meat” and “meats”, “flavour” and “flavor”, and so on, so we standardized the keywords to avoid meaningless relationships. We were then left with 1354 keywords for the next step of the analysis.

Table 6 shows the top 20 authors’ keywords listed in order of the number of occurrences, and classified according to the object of the study, the method and instrument used, the research topic, and the type of meat analyzed. 

“Volatile organic compounds” is the core concept of this review, so it is not surprising that it was also the keyword most frequently used by the authors. “Odor”, “Flavor” and “Sensory” were the most frequent keywords related to VOCs, and of these, odor depends directly on the individual VOC in question, while flavor is a comprehensive indicator combining “odor”, taste and other sensory characteristics [66]. The authors’ keywords also included “color” and the general term “meat quality”. 

Among the keywords related to the methodologies and/or instruments used for the analyses, “SPME-GC-MS” and “GC-MS” were the most frequent [10], followed by GC-O (Song et al., 2018) [67], and E-nose (Shi et al., 2018) [68]. 

The table also shows the major research topics of the articles on VOC profiles: “lipid oxidation” is the most frequent keyword, followed by “irradiation”, “fatty acids”, “and “spoilage”. Lastly, regarding the product analyzed, aside from the general term “meat”, the keywords in order of frequency were “Beef“ > “Pork” > “Dry-cured ham” > “Lamb” > “Chicken”, whereas “sausage” and “burger” were found much less often. 

**Figure 4:**
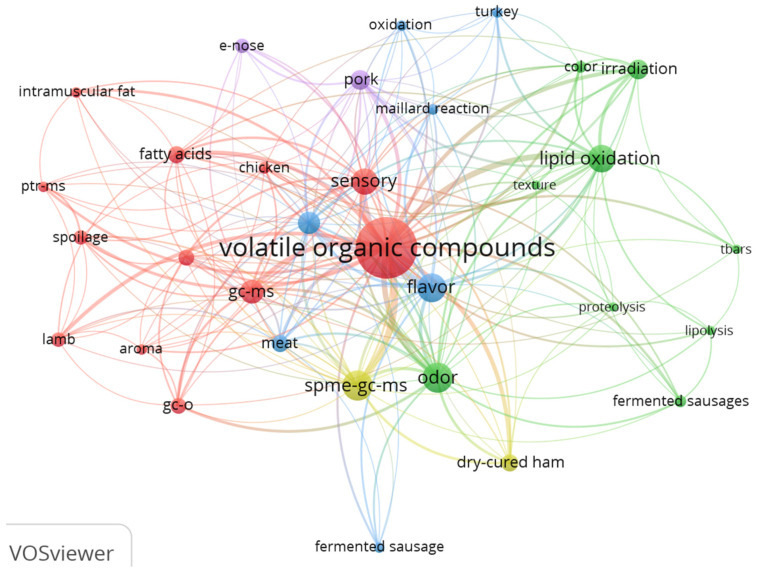
Keyword network for the volatile organic compounds of meat (32 key words with co-occurrences greater than 8 extracted from the 1354 selected authors’ keywords).

In addition to the number of occurrences, we also analyzed the co-occurrences. Figure 4 illustrates the networks of authors’ keyword co-occurrences and shows some interesting associations. 

### 3.7. Types of Meat and Meat Products

Analysis of the authors’ keywords revealed that VOC profiling of meat was mainly focused on two groups of meat products: one was muscle cuts, raw or cooked, obtained from different species and categories of meat animals, and the other was meat products obtained from industrial/artisanal processing. The first group mainly comprised meat cooked by different methods, such as grilling [69], frying [9], microwaving [70], boiling [71], etc. The second group mainly included fermented meat, ham, sausages (pork, horsemeat, poultry) [7], and burgers/patties (from different meats) [31,72]. It is worth noting that little research has been conducted on the effects of different cooking techniques on VOC profiles, so a future research priority should be the interaction between cooking method and the type of meat cooked. There is also little or no research comparing the VOC profiles of raw and cooked meat.

We also classified the articles according to the type of meat studied, and the results were similar but not identical to the ranking of authors’ keywords (Table 6). The highest proportion of all studies were carried out on beef (20%), especially in Europe and America, followed by ham (19%), especially the high-value dry-cured hams typical of Spain and Italy, pork (18%), the most frequently studied meat in China, and sausages (17%). Taking together ham, sausages and pork, the porcine species was the subject of more than half of all studies. The meat from small ruminants (lamb, mutton and goats) was the subject of 10% of all studies, chicken 8%, and other species/products (chicken, turkey, duck, goose, rabbit meat, etc.) the remaining 8%. 

It is worth noting that very few articles deal with more than one type of meat, so the comparison and authentication of species is another research priority. Only one recent article [3] compared the VOC profiles of meat patties obtained from five species/categories (beef, veal, pork, chicken and turkey).

### 3.8. The Evolution of VOC Analytical Methods and Instruments

Aside from meat sampling and preparation, VOC profiling follows three steps: first, detecting the different molecules or fragments of them; second, identifying their chemical nature; and, lastly, associating the identified substance with a corresponding odor through the scientific literature or sensory evaluation by an expert or an olfactory instrument.

**Figure 5:**
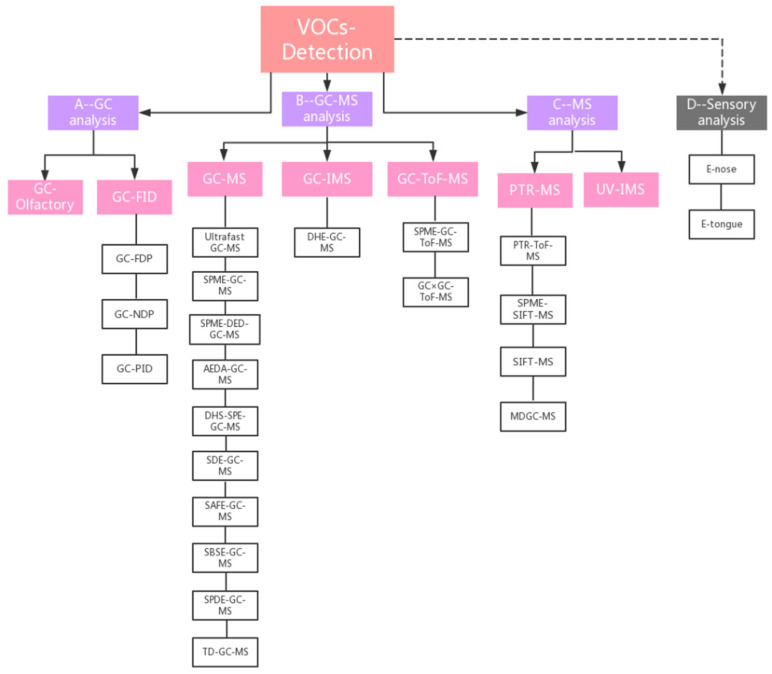
Classification of the instruments/methodologies used for VOC analysis.

The 611 articles selected used several different analytical methods/instruments to detect VOCs. The aim of this review paper is not to provide an analytical description or compare the pros and cons of the different methods, but rather to trace the evolution of the various methods over time and consider the prospects in this field. As the same methods/instruments are often referred to in different articles by different names and/or acronyms, we first had to classify the methods according to analytical principle, and then group them into four major categories. This classification is summarized in Figure 5.

The four categories were gas-chromatographic methods (group A: GC); methods combining gas-chromatography with mass-spectrometry (group B: GC-MS); direct mass-spectrometry methods (group C: MS); and, lastly, sensory methods (group D: sensory analysis), including E-nose and E-tongue.

Table 7 shows the classification of the various methods/instruments and the evolution of their use over the period 2000–2020. 

The first category (A-GC) comprises various gas chromatography (GC) instruments/methods, a technique that has very good separation power [73], and can be combined with various detection techniques for qualitative and quantitative analysis of chemicals. Its major limitation is mainly that it is a very tedious, time-consuming process [74]. Another limitation is that the number of VOCs characterizing meat products can be very high and standards are not always available, so it is not easy to identify the compounds producing the chromatographic peaks. This explains why, with time, these methods/instruments have failed to attract much interest from researchers.

An example of a technique that uses GC but that can be very useful in research on the odor profiles of meat products is GC-O (Olfactory). This was a very popular technique in the early days of meat VOC research because it was a useful aid to experts in identifying odor VOCs, and is often used in combination with other techniques, such as GC-MS, to investigate the relationship between a chemical substance and a certain odor [75]. The main limitation of GC-O/MS is that it cannot provide quantitative measurements. GC-FID is another technique for identifying VOCs [76], which is usually combined with GC-MS and can be optimized for specific volatile compounds [77].

The second category (B-GC-MS) includes methods that combine gas-chromatography for separating the VOCs with mass-spectrometry for characterizing their chemical composition. This category of methods is very successful because it greatly improves the ability to precisely identify VOCs compared with simple GC methods (but is no less time-consuming). The great success of these methods is due in particular to the possibility of combining them with solid-phase-microextraction (SPME-GC-MS), which uses a fiber coated with an extracting phase to capture the VOCs in the headspace of a vial containing the meat sample, avoiding direct contact with the material. This method was used by more than half the researchers and can even now be considered the reference method for VOC analysis. Other interesting methods in this category are GC-IMS [78] and ultrafast GC-MS [79,80,81].

The third group of instruments makes direct use of mass spectrometry (MS) detection methods without using gas-chromatography to separate the VOCs. Some of these methods have been attracting increasing interest in recent years due to their sensitivity and high throughput. PTR-MS [24,82] and PTR-ToF-MS are of particular interest in this regard [18,33,83]. The latter technique was recently combined with SPME-GC-MS to improve the identification of chemical species from mass peak data [84]. 

The fourth group comprises sensory instruments, such as E-nose and E-tongue, that are already commonly used in the field of food safety to detect food-borne pathogens [85]. E-nose is a fast, non-invasive, real-time monitoring tool, consisting of non-selective sensors and a computer program that can discriminate patterns of VOCs, that has been used to analyze several meat samples [86]. Recently, several studies have combined the E-nose instrument with GC-MS to evaluate meat VOCs; these have shown that while E-nose has an excellent ability to discriminate between species, it cannot identify specific chemical substances, so combining it with GC-MS can give better qualitative results [87]. E-tongue is an instrument for evaluating the taste of food, such as sourness, bitterness, and astringency [88], and can be used as a complement to other methods specifically intended for VOC analysis. We found a few papers that used E-tongue in combination with GC-MS to evaluate beef flavor [89,90].

## 4. Conclusions

This bibliometric analysis of articles published in scientific journals on the VOC profiles of meat shed interesting light on the state of research, both past and present. First of all, we found a continuously growing interest in research in this field, and identified the leading journals, authors, institutions and countries involved in it. Most of the research on VOCs is carried out in the USA, particularly on meat products and industry needs, in Southern Europe (Spain and Italy), mainly on food safety and traditional meat products, and in East Asia (China and South Korea), mainly on technological issues and traditional foods. Collaboration network mapping revealed important partnerships, especially between the USA and East Asia, and those of Southern Europe with Northern Europe and Latin America. More than half of the studies were carried out on pork (especially in China), and pork products, such as ham (especially in Spain and Italy), and sausages. Regarding ruminants, almost a quarter of the published research was on beef (especially in the USA and Europe), whereas only about a tenth of all articles dealt with meat and meat products from small ruminants. Avian species and rabbits were much less frequently studied. Numerous different techniques and instruments were also used, and these have evolved over time. Research on VOCs is broadening its objectives, increasingly focusing on food safety, adulteration, and food technology on one side, and authentication of traditional local products, characterization of local breeds and populations, and validating artisanal products, on the other side. Further research which compares meat products from different species and different cooking methods, and the interactions between them, is needed; moreover, it should focus on better characterizing the link between individual VOCs and their perceived odors.

## Figures and Tables

**Table 1:** Summary of the information retrieved and selected for bibliometric analysis.

Items	Results
Timespan	2000 to 2020
Sources (Journals, Proceedings, etc.)	100
Documents selected	611
Citations	14,081
Average citations per document	23.05
Average citations per year per document	2.75
Authors	1862
Documents per author	0.33
Co-authors per document	5.27
Authors’ keywords (DE)	1447

**Table 2:** Ranking of the top 25 of 98 journals that published articles on VOC profiles of meat in 2000–2020 according to the number of articles they published, the quartile in their highest subject category, and their H-index, impact factor and country of publication.

Sources	Articles	Quartile	H-Index	Impact Factor	Country ^1^
*Meat Science*	157	Q1	142	3.483	NL
*Food Chemistry*	60	Q1	221	5.399	NL
*Journal of Food Science*	36	Q1	134	2.081	USA
*Journal of Agricultural and Food Chemistry*	29	Q1	262	3.571	USA
*LWT—Food Science and Technology*	23	Q1	115	3.714	USA
*Food Research International*	18	Q1	134	3.579	NL
*Journal of the Science of Food and Agriculture*	14	Q1	262	3.571	USA
*International Journal of Food Science and Technology*	14	Q2	47	2.383	UK
*Journal of Food Processing and Preservation*	13	Q2	42	1.288	USA
*Food Analytical Methods*	12	Q1	35	2.413	USA
*International Journal of Food Microbiology*	11	Q1	170	4.006	NL
*Poultry Science*	10	Q1	119	1.240	USA
*Journal of Food Science and Technology*	9	Q1	47	1.850	UK
*Radiation Physics and Chemistry*	8	Q2	72	1.984	UK
*Food Control*	8	Q1	103	4.248	NL
*Talanta*	7	Q1	154	4.916	NL
*International Journal of Food Properties*	7	Q2	45	1.398	USA
*Innovative Food Science and Emerging Technologies*	7	Q1	96	3.030	NL
*Food Microbiology*	7	Q1	111	4.089	USA
*Small Ruminant Research*	6	Q2	71	1.210	NL
*Molecules*	6	Q1	131	3.060	CH
*Korean Journal of Food Science and Technology*	6	Q3	18	1.145	KR
*Grasas y Aceites*	6	Q3	43	0.891	ES
*Asian-Australasian Journal of Animal Science*	6	Q1	45	0.530	KR
*Journal of Food Process Engineering*	5	Q2	44	1.448	USA

^1^ ISO Country Codes. NL: Netherlands; USA: United States of America; UK: United Kingdom; KR: South Korea; CH: Switzerland; ES: Spain.

**Table 3:** The top 25 most cited articles, with authors, sources, total citations (TC) and citations per year (TC/year).

Authors	Title	Year	Source	TC	TC/Year
Ercolini D., Russo F., Nasi A., Ferranti P., Villani F. [41]	*Mesophilic and psychrotrophic bacteria from meat and their spoilage potential* in vitro *and in beef*	2009	Appl. Env. Microb. 75(7): 1990–2001.	197	16.42
Casaburi A., Piombino P., Nychas GJ., Villani F., Ercolini D. [42]	*Bacterial populations and the volatilome associated to meat spoilage*	2015	Food Microb. 45: 83–102.	197	32.83
Mielnik M.B., Olsen E., Vogt G. Adeline D., Skrede G. [43]	*Grape seed extract as antioxidant in cooked, cold stored turkey meat*	2006	LWT-Food sci. technol. 39(3): 191–198.	166	11.07
Huang L., Zhao J., Chen Q., Zhang Y. [44]	*Nondestructive measurement of total volatile basic nitrogen (TVB-N) in pork meat by integrating near infrared spectroscopy, computer vision and electronic nose techniques*	2014	Food Chem. 145: 228–236.	155	22.14
Raes K., Balcaen A., Dirinck P., De Winne A., Claeys E., Demeyer D., De Smet S. [45]	*Meat quality, fatty acid composition and flavour analysis in Belgian retail beef*	2003	Meat Sci. 65(4): 1237–1246	152	8.44
Ahn D.U., Jo C., Olson D.G. [46]	*Analysis of volatile components and the sensory characteristics of irradiated raw pork*	2000	Anim. Ind. Report; 1(1)	151	7.19
Brunton N.P., Cronin D.A., Monahan F.J., Durcan R. [35]	*A comparison of solid-phase microextraction (SPME) fibres for measurement of hexanal and pentanal in cooked turkey*	2000	Food Chem. 68(3): 339–345	144	6.86
Ahn D.U., Jo C., Du M., Olson D.G., Nam K.C. [47]	*Quality characteristics of pork patties irradiated and stored in different packaging and storage conditions*	2000	Meat Sci. 56(2): 203–209.	136	6.48
Descalzo A.M., Insani E.M., Biolatto A., Sancho A.M., García P.T., Pensel N.A., Josifovich J.A. [48]	*Influence of pasture or grain-based diets supplemented with vitamin E on antioxidant/oxidative balance of Argentine beef*	2005	Meat Sci. 70(1): 35–44	136	8.50
Olesen P.T., Meyer A.S., Stahnke L.H. [49]	*Generation of flavour compounds in fermented sausages—The influence of curing ingredients, Staphylococcus starter culture and ripening time*	2004	Meat Sci. 66(3): 675–687	124	7.29
Mayr D., Margesin R., Klingsbichel E., Hartungen E., Jenewein D., Schinner F., Märk T.D. [50]	*Rapid detection of meat spoilage by measuring volatile organic compounds by using proton transfer reaction mass spectrometry*	2003	Appl. Envi. Microb. 69(8): 4697–4705	116	6.44
Elmore J.S., Cooper S.L., Enser M., Mottram D.S., Sinclair L.A., Wilkinson R.G., Wood J.D. [51]	*Dietary manipulation of fatty acid composition in lamb meat and its effect on the volatile aroma compounds of grilled lamb*	2005	Meat Sci. 69(2): 233–242.	111	6.94
Martín A., Córdoba J.J., Aranda E., Córdoba M.G., Asensio M.A. [52]	*Contribution of a selected fungal population to the volatile compounds on dry-cured ham*	2006	Intern. J. Food Microb. 110(1): 8–18	109	7.27
Elmore J.S., Warren H.E., Mottram D.S., Scollan N.D., Enser M., Richardson R.I., Wood J.D. [53]	*A comparison of the aroma volatiles and fatty acid compositions of grilled beef muscle from Aberdeen Angus and Holstein-Friesian steers fed diets based on silage or concentrates*	2004	Meat Sci. 68(1): 27–33.	106	6.24
Domínguez R., Gómez M., Fonseca S., Lorenzo J.M. [54]	*Effect of different cooking methods on lipid oxidation and formation of volatile compounds in foal meat*	2014	Meat Sci. 97(2): 223–230.	104	14.86
Nam K.C., Ahn D.U. [55]	*Use of antioxidants to reduce lipid oxidation and off-odor volatiles of irradiated pork homogenates and patties*	2003	Meat Sci. 63(1): 1–8	103	5.72
Moretti V.M., Madonia G., Diaferia C., Mentasti T., Paleari M.A., Panseri S., Pirone G., Gandini G. [56]	*Chemical and microbiological parameters and sensory attributes of a typical Sicilian salami ripened in different conditions*	2004	Meat Sci 66(4): 845–854	101	5.94
Limbo S., Torri L., Sinelli N., Franzetti L., Casiraghi E. [57]	*Evaluation and predictive modeling of shelf life of minced beef stored in high-oxygen modified atmosphere packaging at different temperatures*	2010	Meat Sci. 84(1): 129–136.	99	9.00
Olivares A., Navarro J.L., Flores M. [58]	*Effect of fat content on aroma generation during processing of dry fermented sausages*	2011	Meat Sci. 87(3): 264–273.	98	9.80
Estévez M., Morcuende D., Ventanas S., Cava R. [59]	*Analysis of volatiles in meat from Iberian pigs and lean pigs after refrigeration and cooking by using SPME-GC-MS*	2003	J. Agric. Food Chem. 51(11): 3429–3435.	91	5.06
Nurjuliana M., Che Man Y.B., Mat Hashim D., Mohamed A.K.S. [60]	*Rapid identification of pork for halal authentication using the electronic nose and gas chromatography mass spectrometer with headspace analyzer*	2011	Meat Sci. 88(4): 638–644	91	9.10
Lorenzo J.M., González-Rodríguez R.M., Sánchez M., Amado I.R., Franco D. [61]	*Effects of natural (grape seed and chestnut extract) and synthetic antioxidants (buthylatedhydroxytoluene, BHT) on the physical, chemical, microbiological and sensory characteristics of dry cured sausage “chorizo”*	2013	Food Res. Intern. 54(1): 611–620	91	11.38
Du M., Ahn D.U., Nam K.C., Sell J.L [62]	*Influence of dietary conjugated linoleic acid on volatile profiles, color and lipid oxidation of irradiated raw chicken meat*	2000	Meat Sci. 56(4): 387–395	90	4.29
Elmore J.S., Mottram D.S., Hierro E. [63]	*Two-fibre solid-phase microextraction combined with gas chromatography-mass spectrometry for the analysis of volatile aroma compounds in cooked pork*	2001	J. Chromatog. A 905(1–2): 233–240	89	4.45
Zhou G.H., Zhao G.M [64]	*Biochemical changes during processing of traditional Jinhua ham*	2007	Meat Sci. 77(1): 114–120.	87	6.21

**Table 4:** The most productive authors according to the number of articles published, the H-index and total citations of those articles.

Authors	Country	Institution	Articles	H Index	Citations
Ahn, D.U.	USA	Iowa State University	38	17	1049
Lorenzo, J.M.	Spain	Meat Technology Center of Galicia	37	21	1071
Flores, M.	Spain	Instituto de Agroquímica y Tecnología de Alimentos (IATA-CSIC) Valencia	24	16	651
Lee, E.J.	USA	Iowa State University	15	10	264
Carballo, J.	Spain	Universidad de Vigo	14	11	364
Nam, K.C.	S. Korea	Sunchon National University	13	7	451
Wang, Y.	China	Ningbo University	13	4	64
Jo, C.	USA	Iowa State University	12	7	378
Dominguez, R.	Spain	Meat Technology Center of Galicia	11	7	264
Zhang, Y.	China	Changzhou University	11	5	265
Nunez, M.	Spain	Instituto Nacional de Investigación y Tecnología Agraria y Alimentaria (INIA)	10	9	207
Rivas-Cañedo, A.	Spain	INIA	10	9	214
Liu, Y.	China	Nanjing Agricultural University	9	5	102
Purrinos, L.	Spain	INIA	9	7	208

**Table 5:** The 10 most productive institutions in the field of meat VOC profiles and the numbers of articles published.

Institutions	Articles	Country
Iowa State University	56	USA
Northeast Agricultural University	37	China
Instituto Nacional de Investigación y Tecnología Agraria y Alimentaria (INIA)	21	Spain
Instituto de Agroquímica y Tecnología de Alimentos (IATA-CSIC)	19	Spain
Ningbo University	18	China
Konkuk University	15	South Korea
Universidad de Extremadura	29	Spain
National Institute of Horticultural and Herbal Science Rural Development Administration	14	South Korea
Università degli Studi di Napoli Federico II	13	Italy
Nippon Veterinary and Life Science University	13	Japan

**Table 6:** Occurrences of the authors’ main keywords and keyword networks.

Authors’ Keywords	Occurrences
**Object of study keywords:**	
Volatile organic compounds	304
Odor	73
Flavor	66
Sensory	53
Meat quality	20
Color	12
**Method/instrument keywords:**	
SPME-GC-MS	73
GC-MS	45
GC-O	20
E-nose	18
**Research topic keywords:**	
Lipid oxidation	61
Irradiation	31
Fatty acids	22
Spoilage	16
**Product analyzed keywords:**	
Beef	41
Pork	32
Meat	25
Dry-cured ham	24
Lamb	18
Chicken	11

**Table 7:** Evolution of the use of different methodologies/instruments in research on meat VOC profiles from 2000–2020 and their classification groups.

Instrument	Group	2000–2004	2005–2009	2010–2014	2015–2020	Total
SPME-GC-MS	B-GC-MS	1	14	58	78	151
GC-MS	B-GC-MS	3	5	9	21	38
Olfactory	A-GC	1	3	3	9	16
E-nose	D-sensory	-	-	4	10	14
PTR-MS	C-MS	2	-	1	7	10
GC-FID	A-GC	-	3	1	4	8
Other GC methods	A-GC	-	1	-	2	3
Other GC-MS methods	B-GC-MS	-	6	5	17	28
Other MS methods	C-MS	-	-	4	2	6
Other sensory methods	D-sensory	-	-	-	2	2

## Data Availability

All data were retrieved from the Scopus database.
